# Special Sessions

**DOI:** 10.1111/ene.70676

**Published:** 2026-06-26

## Saturday, June 27 2026

## EAN/Neurotorium: Precision medicine in brain diseases: Biomarkers to personalised care

## SPS01_1

### From empirical to personalized prevention of Parkinson's disease

#### E. Schäffer

##### Christian‐Albrechts‐Universität zu Kiel, Kiel, Germany

## SPS01_2

### AI, biomarkers, and ethical leadership: Transforming neurological procedures towards cognitive health and care

#### I. Haraldsen

##### Oslo University Hospital, Oslo, Norway

## SPS01_3

### Experimental medicine to predict individual differences in drug response: Lessons from dementia

#### J. Rowe

##### Addenbrooke's Hospital/University of Cambridge, Department of Neurology, University of Cambridge Herchel Smith Building Cambridge Biomedical Campus, Cambridge, United Kingdom

## SPS01_4

### Precision medicine in epilepsy

#### D. Pal

##### King's College London, Basic & Clinical Neuroscience, London, United Kingdom

## EAN/EPA: The biological and social determinants of neurological and mental health: Toward a unified brain health perspective

## SPS02_1

### Integrating biological and environmental factors in mental health prevention

#### C. Arango

##### Hospital Universitario La Paz, Chair, Department of Child and Adolescent Psychiatry, Madrid, Spain

## SPS02_2

### Harmonising practices: Bridging biological aspects and social contexts across Europe

#### U. Volpe

##### Unit of Clinical Psychiatry, Department of Clinical Neurosciences/DIMSC, Università Politecnica delle Marche, Ancona, Italy

Achieving sustained improvements in brain and mental health requires approaches that integrate biological knowledge with social, cultural, and systemic determinants of health. Across Europe, advances in neuroscience, genetics, and clinical neurobiology coexist with persistent disparities in mental health outcomes, access to care, and implementation of evidence‐based interventions. These gaps highlight the need for harmonised approaches that connect biological insights with social realities, healthcare systems, and public health strategies.

A unified brain health perspective can be applied across diverse European contexts by addressing interactions between biological vulnerability and social determinants across the lifespan. Biological risk factors—such as neurodevelopmental trajectories, genetic susceptibility, inflammation, and stress‐related neurobiological changes—are shaped by environmental exposure, socioeconomic conditions, education, migration, discrimination, and healthcare access. Understanding these interactions is essential for effective prevention, early intervention, and service planning.

Also, cross‐national differences will be considered in mental health policies, service organisation, and preventive strategies, and how these influence the translation of neuroscientific knowledge into routine practice. Particular attention is given to the challenge of aligning neurobiological advances with population‐level and community‐based interventions, and to the importance of interdisciplinary collaboration across neurology, psychiatry, public health, and social sciences.

Ultimately, advancing brain health in Europe requires preventive, equity‐oriented strategies that integrate biological and social perspectives while remaining adaptable to local contexts.

**Disclosure:** Nothing to disclose.

## SPS02_3

### A child neurology perspective on the origins of neurodevelopmental disorders and their impact in adolescence

#### L. Haataja

##### Department of Pediatric Neurology, Helsinki University Hospital, Helsinki, Finland

Children and adolescents with neurodevelopmental disabilities represent a growing population of individuals with varying functional everyday impairments. These health conditions cause impairments in motor, cognitive, language, behavioral and/or sensory functioning. In 2023, WHO and UNICEF reported that there are approximately 317 million children and adolescents affected by developmental disabilities globally. In the Global Burden of Disease study (2019) the prevalence of these conditions ranged from 7.5% among children under 5 years to 13.9% for those aged 15–19 years.

The origins of neurodevelopmental disorders are diverse and often multifactorial combinations of genetic, epigenetic and environmental factors affect critical periods of neurogenesis and connectivity. Furthermore, adverse perinatal incidents, like preterm birth and birth asphyxia, play a major role in adverse neurodevelopmental outcomes.

Neurodevelopmental disabilities are associated with poor academic outcomes, bullying, loneliness, discrimination, unemployment and somatic health problems like obesity, diabetes, cardiac and respiratory diseases, and mental health conditions. The vicious circle may be accelerated by the fact that the diagnoses of neurodevelopmental disorders are often based on various combinations of symptoms, blurring the line between so‐called typical and atypical development, and, at present, the neuro‐anatomic correlation is to a large degree not detected by the currently available imaging tools in clinics.

There is an immense global need for increasing evidence‐based awareness of neurodevelopmental disorders and developing community‐based strategies for providing opportunities to support individual needs and interventions and secure equal rights to individuals affected by these disorders as members of society.

**Disclosure:** Nothing to disclose.

## MDS‐ES European Basal Ganglia Club

## SPS03_1

### C. David Marsden Award Lecture: What to do if even David Marsden would not have a clue?

#### B. Bloem

##### Radboud Medical Centre, Radboud UMC Center of Expertise for Parkinson & Movement Disorders, Nijmegen, The Netherlands

## SPS03_2

### Dystonia Europe – David Marsden Award Lecture: Striato‐pallidal oscillatory connectivity correlates with symptom severity in dystonia patients

#### R. Lofredi

##### Charité Universitätsmedizin Berlin, Klinik für Neurologie und Experimentelle Neurologie, Berlin, Germany

## Sunday, June 28 2026

## What's new on EAN guidelines?

## SPS04_1

### EAN/ILAE/EpiCare/EPNS clinical practice guidelines in the field of rare and complex epilepsies: Lennox‐Gastaut syndrome (LGS)

#### E. Trinka

##### Uniklinikum Salzburg, Christian‐Doppler‐Klinik, Department of Neurology, Salzburg, Austria

## SPS04_2

### Management of SUNCT/SUNA, paroxysmal hemicrania, and hemicrania continua: Expert EAN consensus recommendations

#### D. Wei

##### King's College Hospital, Department of Basic and Clinical Neuroscience, London, United Kingdom

## SPS04_3

### European guidelines for the symptomatic treatment of atypical Parkinsonian syndromes (MSA, PSP, CBD)

#### C. Palleis

##### LMU University Hospital, Department of Neurology, Munich, Germany

## SPS04_4

### EAN CoCoCare graduation 2025

#### M. Majoie

##### Department of Neurology, Academic Centre for Epileptology, Epilepsy Centre Kempenhaeghe & Maastricht University Medical Centre, Netherlands

EAN CoCoCare Graduation 2025: Training the Next Generation of Cost‐Conscious Neurology Guideline Developers

Marian HJM Majoie Affiliation Department of Neurology, Academic Centre for Epileptology, Epilepsy Centre Kempenhaeghe & Maastricht University Medical Centre, Netherlands

Dauren Ramankulov EAN Scientific Department Coordinator

Clinical guidelines are essential for ensuring high‐quality, cost‐efficient healthcare. They reflect scientific knowledge, guide clinical practices, and identify gaps in research, ultimately improving healthcare outcomes. However, the challenge lies in balancing quality, efficiency, accessibility, and sustainability. In response to this challenge, the Cost‐Conscious Healthcare (CoCoCare) training programme was launched in 2018 at Maastricht University and later adopted by the European Academy of Neurology (EAN) in 2023. This year‐long educational initiative targets neurology residents and early‐career neurologists, equipping them with the skills to develop evidence‐based, economically sustainable clinical guidelines. The programme combines e‐learning, workshops, webinars, self‐directed learning, and practical guideline development, culminating in project presentations at the EAN Congress. Over 140 participants from more than 25 European countries have completed the programme, which has received positive feedback for fostering cost‐conscious decision‐making and promoting active participation in guideline development. CoCoCare's fourth edition, launched at the 2026 EAN Congress, continues to nurture a new generation of experts poised to lead future guideline development within the EAN framework. Graduates gain mentorship, congress grants, and professional recognition, enhancing their careers and contributing to the long‐term sustainability and quality of neurological care across Europe.

**Disclosure:** Nothing to disclose.

## Breakthroughs in treatment neurology – Part 1

## SPS05_2

### Chimeric antigen receptor (CAR) T‐cell therapy in neurological disorders

#### H. Durmus

##### Istanbul Faculty of Medicine, Istanbul, Türkiye

## SPS05_3

### Broad rim lesions as new biomarker for rapid disease progression in Multiple Sclerosis

#### V. Konstantinidis

##### Department of Neurology, Erasmus MC, Rotterdam, The Netherlands

The high diversity in individual clinical disease courses of multiple sclerosis (MS) is likely caused by heterogeneity in proportional contribution of underlying biology. Our understanding of mechanisms driving progressive disease in MS is limited, hampering the development of effective therapies. In our work, we aim to study differentiating underlying mechanisms by extensive biological characterization of people with opposite MS disease trajectories. In the current study, we investigated with an international team MS whiter matter lesion pathology of selected Netherlands Brain Bank (NBB) donors with slow versus rapid MS progression (26 vs. 18 individuals, respectively).

Donors with a rapid disease progression were characterized by high prevalence of a distinct histological MS lesion type marked by a hypocellular demyelinated core surrounded by extensive hypercellular myeloid cell rims. This broad rim lesion (BRL) type was characterized by cellular and transcriptional signatures of innate immune activation, inflammatory cytokine production and apoptosis. Within the entire NBB cohort, presence of BRL associated with rapid disease progression, homozygous carriership of the rs10191329 severity‐associated common genetic risk variant and higher levels of cerebrospinal fluid neurofilament light chain. Myeloid cell fractions in BRL rims stained positive for translocator protein 18‐kDa (TSPO). An independent TSPO positron emission tomography study (114 individuals) revealed variation in white matter lesion myeloid cell rim size, and validated the association between lesions with a broad rim and disability progression in individuals with MS.

Our findings offer crucial insights into mechanisms behind MS progression, identifying BRL as a biomarker for rapid disease progression. Presence of BRL could potentially guide participant selection for future therapeutic trials targeting central nervous system intrinsic inflammation in progressive MS. Overall, our findings demonstrate the potential of translational studies comparing individual disease trajectories to reveal novel aspects of MS biology.

**Disclosure:** Nothing to disclose.

## SPS05_4

### Innovative pharmacological therapies to promote the recovery of patients with disorders of consciousness

#### L. Sanz

##### Service of Neurology, Department of Clinical Neurosciences, CHUV, Lausanne, Switzerland

Disorders of consciousness is a group of neurological conditions resulting from heterogenous etiologies yet with a common clinical phenotype. There are only very scarce therapeutic options at hand to improve clinical outcomes and promote the recovery of consciousness among these patients at the moment, despite the fact that even slim beneficial effects may result in tremendous implications both for the patients' quality of life and the financial burden on healthcare systems. Growing evidence suggests that early interventions may carry the most significant chances of efficacy by targeting brain plasticity and neurotransmitter imbalances shortly after injury, already in the ICU setting. Othman and colleagues have explored the use of pharmacological stimulants (methylphenidate and apomorphine) among patients with disorders of consciousness in the ICU and demonstrated preliminary evidence of increased arousal after the administration of these drugs, which may facilitate later recovery. Pupillary responsiveness was identified as a potential marker of patients responding to these treatments, which may help identify candidates and guide therapy. On a similar note, Alnagger et al. are investigating the use of novel experimental pharmacological agents to enhance recovery of consciousness, using different mechanisms of action. They designed a virtual clinical trial using advanced computational modeling of brain activity to test the effect of psilocybin and lysergic aciddiethylamide (LSD), two psychedelics, on brain dynamics of simulated patients, and demonstrated the potential future role of these innovative treatments in disorders of consciousness.

**Disclosure:** Nothing to disclose.

## Clinical Grand Round

## SPS06_1

### Not your average white matter story

#### R. Vernard‐Valnet

##### Centre Hospitalier Universitaire Vaudoise – CHUV BH07, Department of Clinical, Neuroscience, Lausanne, Switzerland

## SPS06_2

### Beyond the tremor: A path to a hidden diagnosis

#### S. Gehri

##### CHUV (Centre Hospitalier Universitaire Vaudois), Grandson, Switzerland

## SPS06_3

### A seemingly simple head trauma with an unexpected diagnosis

#### P. Lalive

##### Geneva University Hospitals, Geneva, Switzerland

## EAN/ESC: Brain & heart interaction for cognitive function and longevity

## SPS07_1

### Bilateral brain‐heart interaction – An introduction

#### M. Endres

##### Charité Universitätsmedizin Berlin, Department of Neurology and Experimental Neurology, Berlin, Germany

## SPS07_2

### The neuronal system and cardiac function

#### D. Carnevale

##### Sapienza Univeristy of Rome and IRCCS Neuromed, Neuro and Cardiovascular, Pathophysiology, Pozzilli, Italy

## SPS07_4

### Individualized cognitive risk in CV disease

#### R. Proietti

##### University of Liverpool, United Kingdom

## SPS07_5

### Stroke‐heart‐syndrome – An update

#### M. Endres

##### Charité Universitätsmedizin Berlin, Department of Neurology and Experimental Neurology, Berlin, Germany

## Omnipresent stress, its detrimental effects, and the beneficial effects of autogenic training

## SPS08_1

### Physiology and detrimental sequelae of acute and chronic stress, and relaxation techniques reducing noxious stress‐effects

#### M. Hilz

##### University Erlangen‐Nuremberg, Erlangen, Germany

## SPS08_2

### How autogenic training induces autonomic nervous system changes that cause relaxation and comfort

#### M. Hilz

##### University Erlangen‐Nuremberg, Erlangen, Germany

## SPS08_3

### Condensed introductory autogenic training session

#### M. Hilz

##### University Erlangen‐Nuremberg, Erlangen, Germany

## Monday, June 29 2026

## EJON: Emerging technologies driving the next generation of neurological biomarkers

## SPS09_1

### Between hope and uncertainty: Biomarker‐led early diagnosis in neurodegenerative disease

#### P. Scheltens

##### Amsterdam University Medical Centers, Alzheimer Center, Amstelveen, The Netherlands

## SPS09_2

### In search of certainty: Biomarkers and the reproducibility challenge in polyneuropathies

#### A. Roos

##### University Medicine Essen, Department of Pediatric Neurology, Essen, Germany

## SPS09_3

### Neurotechnology and biomarkers for precision diagnosis and prognosis in epilepsy

#### K. Vonck

##### Ghent University Hospital, Department of Neurology, Ghent, Belgium

## SPS10_1

### Posterior fossa stroke: Update on thrombolysis and thrombectomy

#### P. Michel

##### Centre Hospitalier Universitaire Vaudoise – CHUV BH13, Neurology Service, Lausanne Switzerland

## SPS10_2

### Antiepileptogenesis within reach: Preventing epilepsy after stroke

#### E. Trinka

##### Uniklinikum Salzburg, Christian‐Doppler‐Klinik, Department of Neurology, Salzburg, Austria

## Breakthroughs in treatment neurology – Part 2

## SPS10_3

### Disease‐modifying therapies in frontotemporal dementia: Breakthrough clinical trials and emerging treatment paradigms

#### R. Vandenberghe

##### University Hospitals Leuven, Leuven, Belgium

The term “frontotemporal dementia” (FTD) covers a variety of rare diseases with distinct phenotypes, etiologies, and neuropathological features. All FTD diseases have in common that there is no symptomatic or disease‐modifying therapy of proven efficacy and no in vivo biomarkers that accurately reflect the underlying neuropathological hallmark lesions. Current trials mostly target the forms with known genetic causes where the disease mechanism is clear. Progranulin (PGRN) mutations lead to a loss of function with a decrease in PGRN protein levels. At the time of writing, more than five industry‐sponsored trials are ongoing in FTLD due to PGRN mutations. The main mechanisms of action under study are PGRN gene replacement via various delivery modes and PGRN protein substitution. We will review the therapeutic strategies, the opportunities and challenges. For the two other main genetic causes (c9orf72 and MAPT) the therapeutic path forward is currently less clear for various reasons. In sporadic FTLD, an investigator‐initiated study of a myeloperoxidase (MPO) inhibitor, verdiperstat, is ongoing in the semantic variant of primary progressive aphasia.

Progress in clinical development of novel therapies in FTLD also depends on readiness of clinical trial networks organized around hubs at a transnational, European level. Furthermore, due to the historical absence of treatment options, persons who are potentially interested to participate in trials may not have received a diagnosis or may not receive the information. The search for novel therapies for FTLD has intensified over the past five years offering hope to patients and families.

**Disclosure:** RV's institution has a clinical trial agreement (RV as PI) with AviadoBio, BMS, Denali, EliLilly, J&J, Merck, and UCB. RV's institution has a consultancy agreement (RV as chair or member of Data Safety Monitoring board or Data Monitoring Committee) with AC Immune and Novartis. RV's institution has a consultancy agreement (RV as international Advisory Board Member) with Roche. RV's research has been sponsored by the Mady Browaeys Fonds voor Onderzoek naar Frontotemporale Degeneratie.

## The neurology of a changing planet

## SPS11_1

### Effects of heat on human physiology – A perspective on climate change and human resilience

#### H. Pallubinsky

##### Department of Nutrition and Human Movement Sciences, NUTRIM Institute for Nutrition and Translational Research in Metabolism, Maastricht University, the Netherlands

According to the World Health Organization (WHO), heat is among the most dangerous of natural hazards for humans. Projections anticipate the death toll to rise sharply in the upcoming decades, unless effective prevention plans and long‐term strategies are implemented on a wide scale. Understanding how heat affects the human body, who is most vulnerable, and how resilience can be strengthened is therefore critical.

The effects of heat exposure on the human body are dependent on environmental parameters (e.g., ambient temperature, solar radiation, humidity, air velocity), duration of exposure, and individual characteristics such as sex, age, fitness, and health status. Ageing and chronic disease, such as cardiovascular diseases or type 2 diabetes, increase vulnerability to heat strain. Moreover, people living with certain neurological conditions, such as epilepsy, may be negatively affected by climate change, as seizures may be triggered by heat stress.

However, importantly, heat should not be viewed solely as a threat. Repeated exposure induces a range of thermophysiological adaptations, including enhanced sweating, improved skin blood flow, reduced core temperature, and greater cardiovascular stability. Heat therapy, or heat training, has also been shown to induce health benefits, such as enhanced cardiovascular function and improved glucose homeostasis. This talk presents heat as a double‐edged sword. It argues for a shift toward individualized and context‐aware strategies that both protect vulnerable populations and harness the adaptive potential of heat to strengthen human resilience and reduce health risks.

**Disclosure:** None.

## SPS11_2

### Environmental neuroscience: Insights from experimental models and human tissue

#### E. Aronica

##### Department of (Neuro)pathology, Amsterdam UMC Location University of Amsterdam, Amsterdam Neuroscience, Amsterdam, The Netherlands

Climate change and environmental degradation are critical determinants of brain health. Heat stress, air pollution, chemical toxicants, and other environmental exposures increase the risk and progression of neurological disorders. Environmental Neuroscience integrates epidemiology, experimental models, and human tissue studies to uncover how these exposures affect neural structure and function.

The neural exposome, reflecting cumulative exposures across the lifespan, provides a framework to understand how physical, chemical, biological, and social stressors shape neurological vulnerability. Exposures trigger oxidative stress, inflammation, blood–brain barrier disruption, metabolic imbalance, and epigenetic modulation, converging on networks that regulate neuronal excitability, glial activation, and neurovascular integrity.

Experimental approaches, including in vitro systems, organ‐on‐chip platforms, and in vivo models, allow controlled investigation of heat, particulate matter, and chemical exposures. Multi‐tier strategies, from computational screening to rodent models, assess dose dependency, interactions, and gene–environment effects. Human brain tissue studies confirm exposure accumulation and validate mechanisms.

A key convergent pathway involves hypothalamic thermoregulation. Warm‐sensitive neurons and ion channels (TRPV1, TRPC4, SCN1A/Nav1.1) maintain thermal homeostasis; dysfunction increases neuronal excitability, seizure susceptibility, and promotes glial activation, extracellular matrix remodeling, and inflammatory signaling. These mechanisms occur across neurological conditions, including neurodegenerative disorders and epilepsy.

Understanding these pathways is essential for identifying vulnerable populations and guiding preventive and therapeutic strategies. Environmental Neuroscience provides a vital framework to mitigate climate‐related neurological risk.

**Disclosure:** Nothing to disclose.

## SPS11_3

### Adapting neurological care to environmental change

#### S. Sisodiya

##### The National Hospital for Neurology and Neurosurgery, Epilepsy Department, London, United Kingdom

## SPS11_4

### Transforming healthcare systems for a climate‐resilient future

#### D. Campbell‐Lendrum

##### World Health Organization, Geneva, Switzerland

## Brief summary of stress and its sequelae and intensified autogenic training sessions

## SPS12_1

### Brief repetition of the mechanisms during stress and the methods of autogenic training that mitigate the autonomic imbalance induced by acute and chronic stress

#### M. Hilz

##### University Erlangen‐Nuremberg, Erlangen, Germany

## SPS12_2

### Intensified autogenic training session 1

#### M. Hilz

##### University Erlangen‐Nuremberg, Erlangen, Germany

## SPS12_3

### Intensified autogenic training session 2 and summary

#### M. Hilz

##### University Erlangen‐Nuremberg, Erlangen, Germany

## Partnering for the brain in Europe and beyond

## SPS13_1

### Global brain health: iGAP and beyond

## SPS13_2

### Panel 1: Reducing the burden of neurological disorders in Europe: Focus on prevention

## SPS13_3

### Panel 2: Global advocacy for neurology: How to join forces?

